# Learning That Circumcision Is Protective against HIV: Risk Compensation among Men and Women in Cape Town, South Africa

**DOI:** 10.1371/journal.pone.0040753

**Published:** 2012-07-19

**Authors:** Brendan Maughan-Brown, Atheendar S. Venkataramani

**Affiliations:** 1 The Southern Africa Labour and Development Research Unit, University of Cape Town, Cape Town, Western Cape, South Africa; 2 Department of Medicine, Massachusetts General Hospital, MGH Charlestown HealthCare Center, Charlestown, Massachusetts, United States of America; University of Ottawa, Canada

## Abstract

**Objectives:**

We examined whether knowledge of the HIV-protective benefits of male circumcision (MC) led to risk compensating behavior in a traditionally circumcising population in South Africa. We extend the current literature by examining risk compensation among women, which has hitherto been unexplored.

**Methods:**

We used data on Xhosa men and women from the 2009 Cape Area Panel Study. Respondents were asked if they had heard that MC reduces a man’s risk of contracting HIV, about their perceived risk of contracting HIV, and condom use. For each gender group we assessed whether risk perception and condom use differed by knowledge of the protective benefits of MC using bivariate and then multivariate models controlling for demographic characteristics, HIV knowledge/beliefs, and previous sexual behaviors. In a further check for confounding, we used data from the 2005 wave to assess whether individuals who would eventually become informed about the protective benefits of circumcision were already different in terms of HIV risk perception and condom use.

**Results:**

34% of men (n = 453) and 27% of women (n = 690) had heard that circumcision reduces a man’s risk of HIV infection. Informed men perceived slightly higher risk of contracting HIV and were more likely to use condoms at last sex (p<0.10). Informed women perceived lower HIV risk (p<0.05), were less likely to use condoms both at last sex (p<0.10) and more generally (p<0.01), and more likely to forego condoms with partners of positive or unknown serostatus (p<0.01). The results were robust to covariate adjustment, excluding people living with HIV, and accounting for risk perceptions and condom use in 2005.

**Conclusions:**

We find evidence consistent with risk compensation among women but not men. Further attention should be paid to the role of new information regarding MC, and drivers of HIV risk more broadly, in modulating sexual behavior among women.

## Introduction

Randomized clinical trials (RCTs) have shown that medical male circumcision substantially reduces the risk of contracting HIV [1], [2], [3], [4], leading many countries to adopt large-scale male circumcision (MC) campaigns as a strategy to prevent heterosexually acquired HIV infection in men [5]. However, some argue that MC scale-up programs may not confer “real world” impacts, citing evidence from recent African Demographic and Health Surveys showing a lack of association between circumcision and HIV status [6], [7].

While the discrepancy between the observational and experimental evidence could be due to failure to account for heterogeneity in circumcision practices in the former [8], another mechanism that could generate this pattern is risk compensation [9]. That is, those benefitting from MC may reduce protective behaviors such as using condoms because of lower perceived risks of acquiring HIV [10]. The effects of risk compensation on the basis of new information about the protective benefits of MC may be even larger in traditionally circumcising populations, where a non-trivial proportion of circumcisions (typically done by traditional surgeons) result in incomplete foreskin removal, thereby reducing the HIV-protective benefits of the practice [8], and where circumcisions are generally not packaged with risk reduction counseling.

Studies of sexual behavior among medically circumcised men show little or no evidence of risk compensation [11]. Indeed, of the three MC clinical trials, only the South African study showed any change in sexual behavior, with an increase in the number of sexual acts among circumcised men; there were no significant changes in unprotected sex or number of sexual partners [1]. However, these studies did not inform participants about the relationship between MC and HIV, and individuals were not asked about their beliefs regarding this. Furthermore, participants received intensive HIV risk reduction education, which is unlikely to be replicated to that degree by medical MC services.

Studies conducted in traditionally circumcising populations after the 2006 publication of the RCTs, when the link between circumcision and HIV became rigorously established and heavily publicized, yield mixed results. While an observational study on a cohort of men in Cape Town showed evidence of risk compensation among those who had heard about the protective benefits of MC [12], a field experiment in Malawi, where men were randomized to that same information, showed no change in the behavior of previously circumcised men (while uncircumcised men actually adopted safer sexual behaviors) [13].

None of these studies have examined risk compensation among women. Recent work has demonstrated that women alter partner choice in response to information on the prevalence of HIV by age [14]. One can easily imagine that responses to risk information can extend to circumcision, as well. For example, women who are informed about the HIV protective benefits of circumcision may perceive that their potential male partner has a lower probability of being HIV positive, and thereby be more likely to engage in risky sexual behaviors. Risk responses may also occur if women believe that circumcisions also reduce male-to-female transmission of HIV, which is possible given that evidence to the contrary materialized only recently [15]. Importantly, behavioral responses among women based on information about male circumcision may currently be more salient in traditionally circumcision populations, such as the Xhosa, because the vast majority of men are circumcised and women have strong preferences for circumcised men [16].

This study examines whether the acquisition of knowledge regarding the potential HIV-protective effects of MC was associated with a lower perceived risk of contracting HIV and reduced condom use in a sample of Xhosa individuals in Cape Town, South Africa. We extend the literature by also examining risk compensation among women. In addition, we utilized longitudinal information that takes advantage of the timing of the publication of the circumcision RCTs to address the issue of confounding beyond the usual covariate adjustment.

## Methods

### Data

We used data from the Cape Area Panel Study (CAPS). The first wave of CAPS (in 2002) surveyed a representative sample of 4,752 young adults living in Cape Town. A two-stage sample was used, stratified by the three main population groups (black, colored and white). In the first stage, clusters were selected categorized by predominant population group; in the second, households were randomly selected from clusters to achieve a representative sample. Respondents were re-interviewed up to four more times in 2003/2004, 2005, 2006, and most recently in 2009 (wave 5), with the cohort then aged 20–30. The sample initially comprised 2152 blacks and 1328 (62%) were re-interviewed in 2009. Ethical approval was granted by the University of Cape Town and University of Michigan.

We use the 2009 survey wave for the bulk of our analysis. This wave was fielded several years after the circumcision RCT results were publicized but just before the publication of evidence that female partners may not benefit from medical MC. Thus, it represents a period where we would expect to observe risk compensation, if it did occur, among both men and women. In addition, the scope for risk compensation in this sample was high as nearly all men were traditionally circumcised [8].

Our main independent variable was a binary indicator created from responses to the question “Have you ever heard that removing a man’s foreskin reduces the risk of him getting HIV?” (1 =  yes, 0 =  no). Although this question does not use the term “circumcision” it was asked towards the end of a module on MC and it is therefore assumed that participants understood “removing a man’s foreskin” as circumcision.

Our main outcomes of interest were measures of HIV risk perception and sexual behaviors. For the former, respondents were asked: “Do you think you have no risk, a small risk, a moderate risk or a great risk of getting the AIDS virus?” We created an ordinal variable (0–3) with those reporting no perceived risk assigned 0.

In terms of sexual behavior, we created binary variables ( = 1) if the individual reported using a condom at last sex and always/usually using condoms with the most recent partner, respectively. Note, relatively few respondents (men: 8%, women: 5%) reported “usually” using condoms with their most recent partner reported in 2009: all results were substantively similar (i.e. the same level of significance and only minimal differences in coefficients) when analyses were repeated with these individuals recoded into the base category (i.e. not included with those who “always” used condoms).

The final sexual behavior variable (“riskier unprotected sex”) was based on the rationale that inconsistent condom use does not necessarily represent risky sexual behavior (e.g., if both partners are HIV negative and aware of each others’ HIV status). This variable was created to represent individuals who reported not always using condoms with their most recent partner *and* reported their partner’s HIV status as positive or unknown. It is likely that some, or even many, respondents will think their partner does not have HIV and be wrong, which means that in practice having unprotected sex with a partner believed to be HIV negative does not equate to zero risk of contracting HIV. However, having unprotected sex with someone of unknown HIV status should, on average, be perceived as riskier sexual behavior than unprotected sex with a partner believed not to have HIV. Behaviors such as this may be especially sensitive to new information that changes perceptions about how risky such behaviors are.

In terms of control variables, CAPS collected a range of information on sexual behaviors/partners, HIV knowledge, and socioeconomic indicators that could jointly influence perceptions of HIV risk and reported condom use and knowledge about the protective benefits of circumcision. Regarding sexual behaviors/partners, we included binary measures of whether the respondent ever had a sexually transmitted disease, reported that either they or their last partner had concurrent partners, and was currently married, as well as a continuous measure of age at first sex. We also included a measure of HIV knowledge and whether or not the respondent knew someone who died of HIV. The HIV knowledge measure counted correct answers to whether HIV can be transmitted via food prepared by someone with HIV/AIDS, by being coughed or sneezed on, from mother to child, and whether it is possible for a healthy-looking person to have HIV. Furthermore we created an indicator of conspiracy beliefs (equal to 1 if the respondent agreed that HIV was created by humans, AIDS was created by scientists in America, or that AIDS was invented to kill black people), which could influence both risk perceptions and risky sexual practices in so far as they imply skepticism of HIV science and associated prevention messages [17]. Skepticism of HIV science could similarly undermine beliefs about MC reducing the risk of female-to-male HIV transmission. For the socioeconomic controls, we used continuous measures of respondent age, (logged) per capita household income and years of education completed.

Finally, we restricted our sample to men (98%) and women (99%) who reported at least one past sexual encounter. As uncircumcised men may have reacted differently to information about circumcision and given our sample comprised relatively few uncircumcised men (n = 43; 8%), we further restricted our male sample to those who were circumcised by 2009.

### Analysis

We first analyzed descriptive statistics for the outcome and control variables by whether or not the respondent had heard that foreskin removal confers protection against HIV for men. This and all subsequent analyses were conducted separately by gender.

We then estimated multivariate regression models with perceived HIV risk, condom use at last sex, condom use (always/usually) with most recent partner, and reporting inconsistent condom use with a partner whose HIV status is known to be positive or unknown as our main dependent variables. In all models, having heard whether foreskin removal was HIV protective was our main independent variable. All models controlled for the sexual behavior, HIV knowledge and socioeconomic variables described above.

Ordinary Least Squares (OLS) regression was used to estimate models for perceived HIV risk. Probit models were used to estimate the sexual behavior regressions given the discrete nature of these data. For the latter, we present marginal effects as these are more easily interpretable than the coefficients (for a continuous variable, the coefficient reflects the percentage point increase in the probability of observing the dependent variable for a 1 unit change in the independent variable; for binary variables, it reflects a similar change in the dependent variable from moving from 0 to 1 on the independent variable of interest). All standard errors were corrected for heteroskedasticity.

Despite covariate adjustment, it is still possible that unobserved confounders might have driven associations between having heard that MC is protective and HIV risk perceptions and sexual behavior. For example, HIV risk perception and sexual behavior, prior to when information on the protective benefits of circumcision became available, might have been different among the individuals who ended up hearing that male circumcision reduces female-to-male HIV transmission. To assess this, we used data from the 2005 CAPS wave, which was conducted before the results of the male circumcision RCTs were published. While respondents were not asked about their knowledge about the protective effects of circumcision in this wave, they were asked the same risk perception and sexual behavior questions as in the 2009 wave. We conducted tests of the equality of proportions and means for these variables across those who were and were not informed about the HIV protective effects of circumcision in 2009. We also included the 2005 risk perception and sexual behavior variables in the models for the 2009 outcomes. Significant pre-RCT differences in risk perception or sexual behavior or reductions in the coefficient estimates for the 2009 versions of these variables would increase the suspicion of unobserved confounders driving the results.

Our implicit assumption here was that respondents only learned about the link between circumcision and HIV between 2005 and 2009. However, there is some data that suggests that some individuals may have believed this association to be true even before 2005 [18], [19]. Regardless, we believed 2005 to be a reasonable baseline for this study for two reasons. First, it is likely that most would have heard this information after 2005, when it was more widely disseminated. Second, the RCTs results should have made this information more believable and thereby more likely to influence sexual behavior after 2005.

We then assessed the robustness of our findings excluding (a) those individuals who reported being HIV positive at the time of the 2009 interview, and (b) those individuals who tested HIV positive (and who reported previously being tested) during CAPS fieldwork [8]. One reason for this is that people living with HIV may be more knowledgeable about HIV related issues such as the protective benefits of circumcision and more likely to adopt safer sexual practices. Including these individuals could therefore bias our results away from finding a risk compensation effect.

All analyses were conducted with Stata 12.0 (Stata Corporation, College Station, Texas, United States of America).

## Results

*Table 1* presents sample proportions and means. Thirty-four percent of men and 27% of women reported hearing that MC reduces the risk of HIV transmission. Self-perceived risks of acquiring HIV were higher for women (1.25 versus 0.82 for men). Men were more likely to report safe sex than women, in terms of condom use at last sex (75% to 60%) and inconsistent condom use with partners whose HIV status was positive or unknown by respondents (19% versus 31%). Men and women were of similar age and education, but more men reported higher household income and employment rates. Women had higher levels of HIV knowledge and were less likely to hold conspiracy beliefs. They attained sexual debut a full year later than men, were less likely to be engaged in a concurrent sexual network, and were more likely to be married.

**Table 1:** Sample descriptive statistics.

	Men		Women	
	Mean(SD)	N	Mean (SD)	N
***Heard Male Circumcision is Protective***	0.34̂	453	0.27	690
**HIV Risk Perception and Sexual Behavior**				
Perception of HIV risk (0–3)	0.82 (0.85)	394	1.25 (1.01)	595
Used Condom at Last Sex	0.75	440	0.60	672
Always/Usually Used Condoms	0.51	453	0.48	690
Riskier Unprotected Sex	0.19	447	0.31	672
**Demographic and Socioeconomic**				
Age (years)	24.9 (2.6)	453	24.7 (2.6)	690
Education (years)	10.5 (2.0)	453	10.64 (1.7)	690
Per Capita Monthly Household Income(Rand)	969 (1195)	453	726 (851)	690
Employed	0.49	453	0.41	690
Currently Married	0.04	453	0.13	690
Age First Sex (years)	15.5 (1.9)	453	16.6 (1.7)	690
Previous STD	0.28	453	0.25	690
Concurrent Sexual Network	0.43	453	0.25	690
Knows Someone Who Died of HIV	0.63	453	0.58	690
HIV Knowledge Score (0–4)	3.09 (0.86)	453	3.27 (0.75)	690
HIV Conspiracy Belief	0.34	453	0.22	690

Notes: Standard deviations in parentheses.

∧The mean of binary variables – figures without a reported standard deviation – are converted to percentages by multiplying by 100: i.e. 0.34 represents 34%.

Unless otherwise indicated next to variable name, all variables are binary indicators.

Perception of HIV risk is a 0–3 scale with 0 representing no risk and 3 great risk.

Perception of HIV risk is a 0–3 scale with 0 representing no risk and 3 great risk.

Used condom at Last Sex  = 1 if the individual used a condom during the last sexual encounter.

Always/Usually Used Condoms  = 1 if the respondent reported usually or always using condoms with his/her most recent sexual partner.

Riskier Unprotected Sex  = 1 if the individual reported never or sometimes using condoms with a partner reported to be of.

unknown or positive HIV serostatus.

Per Capita Monthly Household Income is provided in Rand. During the time of survey, the exchange rate was 1 Rand  = 0.129 USD.

Previous STD refers to whether the individual had a known STD in the past or reported symptoms of dysuria, genital.

discharge, or genital sores at any time.

Concurrent Sexual Network  = 1 if the respondent reported having a concurrent sexual partner or if he/she reported that.

the last partner was in a concurrent sexual relationship, as well.

HIV Knowledge Score counted correct answers to whether HIV can be transmitted via food prepared by someone with.

HIV/AIDS, by being coughed/sneezed on, from mother to child, and whether it is possible for a healthy-looking person to have HIV.

HIV Conspiracy Belief  = 1 if the responded believed that HIV was created by humans, AIDS was created by scientists America, or that AIDS was invented to kill blacks.

*Table 2* displays comparisons of the outcome and control variables by gender and whether or not the respondent had heard that MC reduces female-to-male HIV transmission. Among men, informed respondents were more likely to report being at risk for HIV (p<0.05) and more likely to report condom use at last sex (p<0.01), a pattern that is opposite of risk compensation. In terms of the covariates, informed men had greater HIV knowledge and were far less likely to believe in HIV conspiracy theories. Differences in the other variables were not statistically significant.

**Table 2:** Descriptive statistics by whether respondent had heard that MC was partially protective against HIV.

	Men					Women				
	Did Not Hear MC Protective	N	Heard MC Protective	N	P-value of Difference	Did Not Hear MC Protective	N	Heard MC Protective	N	P-value of Difference
**HIV Risk Perception and Sexual Behavior**										
Perception of HIV risk	0.73 (0.83)	248	0.96 (0.88)	146	0.011	1.31 (1.04)	436	1.08 (0.94)	159	0.014
Used Condom at Last Sex	0.71	288	0.84	152	0.002	0.62	494	0.54	178	0.055
Always/Usually Used Condoms	0.50	297	0.53	156	0.539	0.51	504	0.39	186	0.008
Riskier Unprotected Sex	0.21	294	0.16	153	0.146	0.27	494	0.44	178	0.000
**Demographics & Socioeconomic Status**										
Age	25.0 (2.7)	297	24.7 (2.4)	156	0.321	24.7 (2.6)	504	24.9 (2.6)	186	0.311
Education	10.4 (2.0)	297	10.7 (1.8)	156	0.199	10.6 (1.7)	504	10.7 (1.6)	186	0.792
Logged Per Capita Monthly Household Income	6.44 (0.90)	297	6.48 (0.950)	156	0.658	6.21 (0.9)	504	6.18 (0.9)	186	0.683
Employed	0.50	297	0.47	156	0.628	0.41	504	0.38	186	0.434
Currently Married	0.04	297	0.04	156	0.822	0.13	504	0.14	186	0.815
**Other Covariates**										
Age First Sex	15.5 (1.88)	297	15.5 (1.81)	156	0.799	16.57 (1.68)	504	16.67 (1.65)	186	0.493
Previous STD	0.26	297	0.30	156	0.382	0.24	504	0.27	186	0.438
Concurrent Sexual Network	0.41	297	0.46	156	0.333	0.26	504	0.22	186	0.337
Knows Someone Who Died of HIV	0.62	297	0.64	156	0.653	0.57	504	0.61	186	0.344
HIV Knowledge Score	2.92 (0.88)	297	3.43 (0.70)	156	0.000	3.25 (1.68)	504	3.32 (0.73)	186	0.244
HIV Conspiracy Belief	0.40	297	0.21	156	0.000	0.22	504	0.23	186	0.717

Notes: Standard deviation in parentheses.

P-values derived from two-sample differences in means or proportion test across groups.

See Table 1 notes for full details regarding variables.

The pattern for women is markedly different. Informed women reported, on average, a lower perception of risk of getting HIV (p<0.05), were less likely to report condom use at last sex (p<0.10) and always/usually using condoms with the most recent partner (p<0.01), and more likely to report inconsistent condom use with an HIV-positive or unknown serostatus partner (p<0.01). Importantly, we found no significant differences in any of the control variables for women, indicating that women who had heard that MC reduces HIV transmission were similar to those who had not heard this information. These bivariate results of the relationship between being informed about the HIV protective benefits of MC and risk perceptions and sexual behaviors were borne out in the regression analyses as well.

*Table 3* presents regression estimates for men. Being informed about the protective benefits of MC was associated with a higher perception of HIV risk (p<0.10) and a higher likelihood of using condoms at last sex (p<0.10). In contrast, the estimates for women (*Table* 4) illustrate that those informed of the MC-HIV risk link perceived themselves at lower risk of getting HIV (p<0.01), were 7.9 (p<0.10) and 11.6 (p<0.01) percentage points less likely to have used condoms at last sex or report always/usually using condoms with most recent partner, respectively, and 17.9 percentage points more likely to report inconsistent condom use with partners of seropositive or unknown HIV status (p<0.01). We examined whether these results differed if we recoded our main independent variable to equal 1 only for those individuals who not only heard that circumcision was protective but also reported believing this (64% of men and 88% of women who had heard, respectively). The results were substantively unchanged (available upon request).

**Table 3:** Adjusted association between hearing MC was protective, HIV risk perceptions and condom use behaviors – men.

	(1)	(2)	(3)	(4)
	OLS	Probit	Probit	Probit
	Perception ofHIV risk (0–3)	Used Condomat Last Sex	Always/usuallyUsed Condoms	Riskier Unprotected Sex
***Heard Male Circumcision is Protective***	0.174*	0.0869*	***−0.00266***	***−0.0673***
	***(0.0926)***	***(0.0453)***	***(0.0534)***	***(0.0418)***
Age	−0.0126	0.00314	−0.0249**	0.000650
	(0.0177)	(0.00832)	(0.00994)	(0.00728)
Education	−0.0155	0.00723	0.0197	−0.0279***
	(0.0217)	(0.0109)	(0.0129)	(0.0103)
Logged Per Capita Monthly Household Income	0.0291	0.0276	0.0448	−0.0122
	(0.0505)	(0.0235)	(0.0273)	(0.0231)
Employed	−0.0484	0.0114	−0.0761	−0.0504
	(0.0868)	(0.0422)	(0.0504)	(0.0398)
Currently Married	−0.109	−0.255**	−0.377***	
	(0.207)	(0.110)	(0.125)	
Age First Sex	−0.0260	0.0143	0.0240*	−0.00107
	(0.0238)	(0.0115)	(0.0142)	(0.0110)
Previous STD	0.300***	0.0298	−0.0621	0.0888**
	(0.0987)	(0.0465)	(0.0559)	(0.0444)
Concurrent Network	0.0422	0.0679	−0.0246	0.111***
	(0.0880)	(0.0427)	(0.0511)	(0.0410)
Knows Someone Who Died of HIV	0.291***	0.0484	−0.102*	−0.0866**
	(0.0874)	(0.0451)	(0.0525)	(0.0423)
HIV Knowledge Score	0.0999*	0.0277	0.0126	0.000321
	(0.0522)	(0.0253)	(0.0318)	(0.0254)
HIV Conspiracy Belief	0.0378	−0.154***	−0.104*	−0.0290
	(0.103)	(0.0473)	(0.0554)	(0.0426)
N	310	350	360	336

Notes: The coefficients for the model for Perception of HIV Risk were estimated using OLS. The coefficients in the models for the condom use variables and Riskier Unprotected Sex reflect probit marginal effects.

Robust standard errors in parentheses.

*** p<0.01, ** p<0.05, * p<0.1.

See Table 1 notes and main text for variable definitions.

**Table 4:** Adjusted associations between hearing was protective, HIV risk perceptions and condom use behaviors – women.

	(1)	(2)	(3)	(4)
	OLS	Probit	Probit	Probit
	Perception ofHIV risk	Used Condomat Last Sex	Always/usuallyUsed Condoms	Riskier Unprotected Sex
***Heard Male Circumcision is Protective***	−0.238***	−0.0792*	−0.116***	0.179***
	(0.0906)	(0.0442)	(0.0439)	(0.0414)
Age	0.0487***	−0.00936	−0.00496	0.0194***
	(0.0169)	(0.00803)	(0.00815)	(0.00745)
Education	−0.0107	0.000849	0.0370***	−0.0476***
	(0.0276)	(0.0125)	(0.0131)	(0.0120)
Logged Per Capita Monthly Household Income	−0.0418	−0.00204	−0.0175	0.0103
	(0.0499)	(0.0244)	(0.0250)	(0.0228)
Employed	−0.112	0.0116	0.0210	−0.0362
	(0.0884)	(0.0431)	(0.0435)	(0.0400)
Currently Married	−0.254*	−0.245***	−0.316***	−0.0829
	(0.133)	(0.0615)	(0.0616)	(0.0539)
Age First Sex	−0.0454*	0.0270**	0.0116	−0.0132
	(0.0263)	(0.0120)	(0.0123)	(0.0113)
Previous STD	−0.0776	0.0151	−0.00386	0.0323
	(0.0968)	(0.0458)	(0.0459)	(0.0431)
Concurrent Network	0.116	0.0967**	−0.000685	−0.0704*
	(0.0868)	(0.0451)	(0.0463)	(0.0413)
Knows Someone Who Died of HIV	0.277***	0.0821**	0.0134	−0.0631*
	(0.0820)	(0.0405)	(0.0409)	(0.0378)
HIV Knowledge Score	−0.0646	−0.0288	−0.0501*	0.00933
	(0.0578)	(0.0267)	(0.0269)	(0.0259)
HIV Conspiracy Belief	0.401***	−0.0947*	0.0446	−0.0609
	(0.119)	(0.0494)	(0.0493)	(0.0450)
N	595	672	690	672

Notes: The coefficients for the model for Perception of HIV Risk were estimated using OLS. The coefficients in the models for the condom use variables and Riskier Unprotected Sex reflect probit marginal effects.

Robust standard errors in parentheses.

*** p<0.01, ** p<0.05, * p<0.1.

See Table 1 notes and main text for variable definitions.

*Table 5* compares HIV risk perceptions and sexual behaviors reported in 2005 across men and women who did and did not report knowledge about the protective benefits of MC in 2009. Most, but not all, respondents from 2009 were surveyed in 2005. For men, the pattern of the risk perception and sexual behavior variables is quite similar to the 2009 data. For women, unlike in 2009, there was no evidence that those who had heard about MC and HIV transmission differed markedly from their counterparts in terms of HIV risk perception or sexual behaviors in 2005, prior to when well-publicized RCT evidence on the protective benefits of circumcision became available. Indeed, in 2005, women who reported being informed of the protective benefits of MC in 2009 actually reported slightly higher personal HIV risks, which was opposite to the finding in 2009. And although informed women were 4 and 6 percentage points less likely to have used condoms during last sex or report always/usually using condoms, respectively, none of these differences were statistically significant. Of note, the inclusion of the 2005 risk perception and sexual behavior variables in the regression models for the 2009 outcomes did not change the substantive results (see Table S1), which indicates that the small differences in risk perception and sexual behavior between our groups of interest in 2005 did not drive our main findings.

**Table 5:** Association between having heard MC was protective in 2009 and HIV risk perception and condom use in 2005.

	Men					Women				
	Did Not Hear MC Protective	N	Heard MC Protective	N	P-value of Difference	Did Not HearMC Protective	N	Heard MC Protective	N	P-value of Difference
**Risk Perception and Sexual Behavior in 2005**										
Perception of HIV risk (0–3)	0.70 (0.90)	230	0.70 (0.84)	129	0.949	0.93 (0.98)	400	1.05 (1.0)	142	0.208
Used Condom at Last Sex	0.74	205	0.83	113	0.065	0.66	381	0.61	137	0.367
Always/usually Used Condoms	0.65	208	0.69	118	0.549	0.48	392	0.42	138	0.210

Notes: Standard deviation in parentheses.

P-values derived from two-sample differences in means or proportion test across groups.

V.

Finally, the magnitude and significance of our results were virtually unchanged with the exclusion of those individuals who reported being HIV positive prior to the interview as well as those who tested positive during CAPS fieldwork and had reported being tested in the past (see Table S2).

## Discussion

Understanding behavioral responses to new information regarding HIV risk is critical for designing effective HIV prevention policies. As medical MC programs continue to expand, knowledge of the protective benefits offered by MC will, too, become more widespread and any potential offsetting impacts from increases in risky sexual behaviors among men and women will need to be accounted for.

Using data from a sample of Xhosa individuals in Cape Town, South Africa, a culture that practices traditional circumcision, we find no evidence of risk compensating behavior among men. However, we found that women who had heard that MC partially protects men against HIV were less likely to perceive being at risk of HIV themselves. Perhaps as a consequence, informed women were also less likely to use condoms and more likely to engage in unprotected sex with partners of positive or unknown serostatus. These associations were substantively and statistically significant, and persisted after controlling for a rich set of covariates for socioeconomic status, sexual behavior and HIV knowledge. We did not find any statistically significant differences in sexual behaviors in 2005 across women who ended up reporting in 2009 that MC was protective versus those who did not, nor did the inclusion of the 2005 variables change the results of the regressions for the 2009 outcomes. As RCT evidence about the protective benefits of MC was likely not widely known in 2005, this finding adds additional confidence that unmeasured confounders, such as pre-existing attitudes or beliefs about sexual behavior and perceived HIV risk, did not drive our results.

A key question then is why was there evidence for behaviors consistent with risk compensation among women but not for men? There are several possibilities. First, as alluded to in the introduction, women may have inferred from new information about the protective benefits of MC that the population prevalence of HIV among men was lower than previously supposed, thereby perhaps obviating the need to consistently practice safe sex. Another possibility is that women may have inferred a lower risk of male-to-female transmission of HIV in addition to the demonstrated lower risk of female-to-male transmission in the RCTs. Even if men also shared this belief, we would expect to find more dramatic evidence of risk compensation for women because of the higher baseline prevalence of HIV among women [20] and, consequently, self-perceived risk of contracting HIV in this group (this follows from Bayes’ theorem).

Perceived changes in prevalence and transmission probabilities owing to MC may also reduce the perceived benefits of bargaining with men for condom use during sex. If women have less power relative to men in sexual relationships [21], bargaining for protected sex may come at significant cost to the relationship due to a negative response from the male partner. Women who have heard that MC protects against HIV transmission may therefore be less likely to take on the risk of a negative response and elect not to use condoms.

It is important to note that that the pattern of risk compensation seen for women may actually reflect the behaviors of men. In our data, the average woman was four years younger than their most recent sexual partner. It is therefore possible that men older than 30, whom a significant proportion of the CAPS women were having sex with, responded in different ways to information about MC than did younger men. We do not observe these older men in our data set, which is also a potential shortcoming of much of the recent literature, as well.

Finally, men and women may differ in terms of opportunities to corroborate and contextualize new information. It is well known that circumcision is one of the most sacred and secretive rites practiced by the Xhosa [16]. In this context, it is frowned upon for women and uncircumcised men to talk about circumcision. Thus, upon hearing that MC may reduce HIV transmission, circumcised men may be able to discuss this further with their peers whereas women may not. The pattern in our data may be generated by men either learning that circumcision does not reduce their HIV risk to zero or having HIV (re)introduce as a salient issue in their day-to-day lives as a result of conversations with peers, while women are unable to have these discussions and potentially come away with a false impression of what MC might mean for their own risk.

Future research should take each of these nuances into account. Along these lines, one limitation of this study was that we lacked the data to truly understand the mechanisms underlying the results. There are several other limitations, as well. Most importantly, our research design does not necessarily account for all unobserved confounders that may have influenced our results. Certainly, the robustness to covariate adjustment, the lack of significant differences in sexual behaviors in the survey wave conducted before the medical MC RCTs were published, and the lack of change in the estimates when these past behaviors were included in the main regression models argue against the importance of unmeasured confounders. However, we continue to urge caution, especially since small differences between informed and uninformed women (though only for condom use and not risk perception) were present even in 2005.

In addition, small sample sizes, the lack of more nuanced questions on how respondents interpreted information regarding the potential protective benefits of MC, and the lack of data on the timing of when knowledge about the latter was obtained in relation to sexual behavior limit our ability to fully understand the dynamics of potential risk compensating behavior. In addition, we do not have data from the present period, where knowledge that MC may not reduce the risk of male-to-female HIV transmission may be more widespread, thereby reducing risk compensating behavior on the part of women.

Addressing these limitations can form the basis of a fruitful research agenda towards developing a full understanding of risk compensation, both in traditionally circumcising populations as well as in those being targeted for medical MC roll-outs. Along these lines, we advocate for prospective studies incorporating larger numbers of women and men that collect data on the extent to which individuals internalize beliefs about both female-to-male and male-to-female transmission of HIV both before and after circumcision as well as detailed information on sexual behavior and HIV test results. Randomization of information regarding MC can be a useful tool for inference in this context. The results from such studies would help expand and refine the policy implications from the present work. In particular, our results point towards the need for risk reduction education efforts among women to counter risk compensation associated with male circumcision.

## Supporting Information

Table S1 **Presents regression results similar to** **Table 3** **and** **4** **but now including sexual behavior and risk perception information from 2005 as additional controls.**
(DOC)

Table S2 **Presents regression estimates restricting the sample to those who tested negative for HIV.**
(DOCX)

## References

[1] Auvert B, Taljaard D, Lagarde E, Sobngwi-Tambekou J, Sitta R (2005). Randomized, Controlled Intervention Trial of Male Circumcision for Reduction of HIV Infection Risk: The ANRS 1265 Trial.. PLoS Medicine.

[2] Bailey RC, Moses S, Parker CB, Agot K, Maclean I (2007). Male Circumcision for HIV prevention in Young Men in Kisumu, Kenya: A Randomized Controlled Trial.. The Lancet.

[3] Gray R, Kigozi G, Serwadda D, Makumbi F, Watya S (2007). Male circumcision for HIV prevention in men in Rakai, Uganda: a randomised trial.. The Lancet.

[4] Siegfried N, Muller M, Deeks JJ, Volmink J (2009). Male Circumcision for Prevention of Heterosexual Acquisition of HIV in Men.. Cochrane Database of Systematic Reviews.

[5] WHO/UNAIDS (2011). Progress In Scale-Up of Male Circumcision for HIV Prevention in Eastern and Southern Africa.. Geneva: WHO Publications.

[6] Green LW, Travis JW, McAllister RG, Peterson KW, Vardanyan AN (2010). Male Circumcision and HIV Prevention: Insufficient Evidence and Neglected External Validity.. American Journal of Preventive Medicine.

[7] Mishra V, Medley A, Hong R, Gu Y, Robey B (2009). Levels and Spread of HIV Seroprevalence and Associated Factors: Evidence from National Surveys.. Calverton, Marlyand, USA: Macro International, Inc.

[8] Maughan-Brown B, Venkataramani AS, Nattrass N, Seekings J, Whiteside AW (2011). A Cut Above the Rest: Traditional Male Circumcision and HIV Risk Among Xhosa Men in Cape Town, South Africa.. Journal of Acquired Immune Deficiency Syndromes.

[9] Cassell M, Halperin D, Shelton J, Stanton D (2006). Risk compensation: the Achilles’ heel of innovations in HIV prevention?. BMJ.

[10] Eaton LA, Kalichman SC (2007). Risk Compensation in HIV Prevention: Implications for Vaccines, Microbicides, and Other Biomedical HIV Prevention Technologies.. Current HIV/AIDS Reports.

[11] Mattson CL, Campbell RT, Bailey RC, Agot K, Ndinya-Achola JO (2008). Risk Compensation is not Associated with Male Circumcision in Kisumu, Kenya: A Multi-Faceted Assessment of Men Enrolled in a Randomized Controlled Trial.. PLoS ONE.

[12] Eaton LA, Cain DN, Agrawal A, Jooste S, Udemans N (2011). The Influence of Male Circumcision for HIV Prevention on Sexual Behavior Among Traditionally Circumcised Men in Cape Town, South Africa.. International Journal of STD and AIDS.

[13] Godlonton S, Munthali A, Thornton R (2011). Behavioral Response to Information? Circumcision, Information, and HIV Prevention.. Bureau for Research and Economic Analysis of Development Working Paper no.

[14] Dupas P (2011). Do Teenagers Respond to HIV Risk Information? Evidence from a Field Experiment in Kenya.. American Economic Journal: Applied Economics.

[15] Wawer MJ, Makumbi F, Kigozi G, Serwadda D, Watya S (2009). Circumcision in HIV-Infected Men and its Effect on HIV Transmission to Female Partners in Rakai, Uganda: A Randomized Controlled Trial.. The Lancet.

[16] Vincent L (2008). ‘Boys Will Be Boys’: Traditional Xhosa Male Circumcision, HIV and Sexual Socialisation in Contemporary South Africa.. Culture, Health and Sexuality.

[17] Grebe E, Nattrass N (2011). AIDS Conspiracy Beliefs and Unsafe Sex in Cape Town.. AIDS and Behavior Epub Before Print: DOI 10.1007/s10461-10011-19958-10462.

[18] Lagarde E, Dirk T, Puren A, Reathe R, Bertran A (2003). Acceptability of Male Circumcision as a Tool for Preventing HIV Infection in a Highly Infected Community in South Africa.. AIDS.

[19] Rain-Taljaard R, Lagarde E, Taljaard D, Campbell C, MacPhail C (2003). Potential for an Intervention Based on Male Circumcision in a South African Town with High Levels of HIV Infection.. AIDS Care.

[20] Actuarial Society of South Africa (2011). ASSA 2008 HIV/AIDS Projection Models. Actuarial Society of South Africa website.. http://aids.actuarialsociety.org.za/Accessed.

[21] Dunkle KL, Jewkes RK, Brown HC, Gray GE, McIntryre JA (2004). Gender-Based Violence, Relationship Power, and Risk of HIV Infection in Women Attending Antenatal Clinics in South Africa.. The Lancet.

