# Supplementary material for: Learning That Circumcision Is Protective against HIV: Risk Compensation among Men and Women in Cape Town, South Africa
Source: PLoS One. 2012 Jul 19;7(7):e40753. doi: 10.1371/journal.pone.0040753 (PMC3400649; doi:10.1371/journal.pone.0040753)
Supplement: Table S1 — Presents regression results similar to Table 3 and 4 but now including sexual behavior and risk perception information from 2005 as additional controls. (DOC) [file pone.0040753.s001.doc]

***Table S1 – Adjusted Association Between Having Heard MC was Protective, Risk Perceptions and Condom Use Behaviors in 2009 Controlling for Risk Perception and Condom Use in 2005***

|  | (1) | (2) | (3) |
| --- | --- | --- | --- |
|  | OLS | Probit | Probit |
|  | Perception of HIV risk (0-3) | Used Condom at Last Sex | Always/usually Used Condoms |
| ***Men*** |  |  |  |
| Heard Male Circumcision is Protective | 0.206* | 0.026 | -0.024 |
|  | (0.120) | (0.053) | (0.068) |
|  |  |  |  |
| N | 245 | 270 | 277 |
|  |  |  |  |
| ***Women*** |  |  |  |
| Heard Male Circumcision is Protective | -0.209** | -0.112** | -0.147*** |
|  | (0.102) | (0.057) | (0.053) |
|  |  |  |  |
| N | 391 | 440 | 452 |

Notes:

-Each cell represents an estimate from a different regression.

-The coefficients for the model for Perception of HIV Risk were estimated using OLS. The coefficients in the models for the condom use variables reflect probit marginal effects.

-Robust standard errors in parentheses.

-*** p<0.01, ** p<0.05, * p<0.1

-Models are the same as those estimated in Tables 3 and 4, except HIV risk perceptions and dummies for condom use at last sex and always using condoms in 2005 are included as additional controls. See Table 3 and 4 for a listing of the other covariates included (not shown here to reduce clutter).
