# Supplementary material for: Learning That Circumcision Is Protective against HIV: Risk Compensation among Men and Women in Cape Town, South Africa
Source: PLoS One. 2012 Jul 19;7(7):e40753. doi: 10.1371/journal.pone.0040753 (PMC3400649; doi:10.1371/journal.pone.0040753)
Supplement: Table S2 — Presents regression estimates restricting the sample to those who tested negative for HIV. (DOCX) [file pone.0040753.s002.docx]

***Table S2 – Adjusted Association Between Having Heard MC was Protective, HIV Risk Perception and Condom Use Behaviors Among Those Who Tested HIV Negative During CAPS 2009***

|  | (1) | (2) | (3) | (4) |
| --- | --- | --- | --- | --- |
|  | OLS | Probit | Probit | Probit |
|  | Perception of HIV risk (0-3) | Used Condom at Last Sex | Always/usually Used Condoms | Riskier Unprotected Sex |
| ***Men*** |  |  |  |  |
| Heard Male Circumcision is Protective | 0.206** | 0.081* | -0.011 | -0.067 |
|  | (0.098) | (0.046) | (0.056) | (0.043) |
|  |  |  |  |  |
| N | 354 | 396 | 408 | 387 |
|  |  |  |  |  |
| ***Women*** |  |  |  |  |
| Heard Male Circumcision is Protective | -0.219** | -0.062 | -0.142*** | 0.206*** |
|  | (0.101) | (0.050) | (0.048) | (0.048) |
|  |  |  |  |  |
| N | 460 | 511 | 521 | 506 |

Notes:

-Each cell represents an estimate from a different regression.

-The coefficients for the model for Perception of HIV Risk were estimated using OLS. The coefficients in the models for the condom use variables reflect probit marginal effects.

-Robust standard errors in parentheses.

-*** p<0.01, ** p<0.05, * p<0.1

-Models are the same as those estimated in Tables 3 and 4. However, here the sample excludes those who tested HIV positive during CAPS fieldwork in 2009 and who were tested for HIV prior to this.

-See Table 3 and 4 for a listing of the other covariates included (not shown here to reduce clutter).
